# Outcomes of patients in a pre-dialysis clinic and implications for shared decision making

**DOI:** 10.1093/ckj/sfaf211

**Published:** 2025-07-08

**Authors:** Hannah O'Keeffe, Rosemary Donne, Philip A Kalra, Ibrahim Ali

**Affiliations:** Renal Department, Salford Royal Hospital, Northern Care Alliance, Manchester, United Kingdom; Faculty of Biology, Medicine and Health, University of Manchester, Manchester, United Kingdom; Renal Department, Salford Royal Hospital, Northern Care Alliance, Manchester, United Kingdom; Renal Department, Salford Royal Hospital, Northern Care Alliance, Manchester, United Kingdom; Faculty of Biology, Medicine and Health, University of Manchester, Manchester, United Kingdom; Renal Department, Salford Royal Hospital, Northern Care Alliance, Manchester, United Kingdom; Faculty of Biology, Medicine and Health, University of Manchester, Manchester, United Kingdom

**Keywords:** advanced kidney care, age, CKD, dialysis, pre-dialysis

## Abstract

**Background:**

The association of end-stage kidney disease (ESKD) with poor outcomes is well recognized. Education and discussions with patients with advanced chronic kidney disease (CKD) are important to facilitate shared decision making regarding care.

**Methods:**

This study reports longitudinal follow-up of all patients who attended the Advanced Kidney Care Service (AKCS) in a tertiary renal centre in the UK. Patients are routinely referred to AKCS once their estimated glomerular filtration rate (eGFR) drops below 20 mL/min/1.73 m^2^. A total of 1957 patients who first attended between September 2011 and September 2018 were included, with a minimum of 5 years follow-up to 30 September 2023.

**Results:**

During follow-up, 55.7% of the cohort commenced renal replacement therapy (RRT), the initial modality was haemodialysis in 57.2%, peritoneal dialysis in 27.3% and a pre-emptive transplant in 15.5%, of which 42.6% were from live donors. Conservative management was chosen by 17.9% of patients. Of those who had opted for RRT, 26.7% died before reaching it. The 5-year survival was 49.6% from first attendance at AKCS. The 5-year survival rates by age group were: <50 years, 84.2%; 50–64 years, 66.1%; 65–79 years, 40.1%; and ≥80 years, 22.3% (*P *< .001). The 5-year survival on haemodialysis was 49.6%, peritoneal dialysis 54.7% and 92.3% for pre-emptive transplant 92.3%. For those over 80 years of age a modest survival benefit was seen with RRT, with a median survival of 17.4 months from RRT commencement, compared with 11.8 months once the eGFR declined below 10 mL/min/1.73 m^2^ in the conservative group.

**Conclusion:**

This study highlights the high competing mortality in an advanced CKD cohort. The high rates of pre-emptive transplantation and peritoneal dialysis initiation demonstrate the benefits of a structured AKCS strategy. Older patients with ESKD, particularly those aged over 80 years, have poor outcomes, regardless of whether they choose RRT or conservative management.

## Graphical Abstract

**Graphical Abstract:**
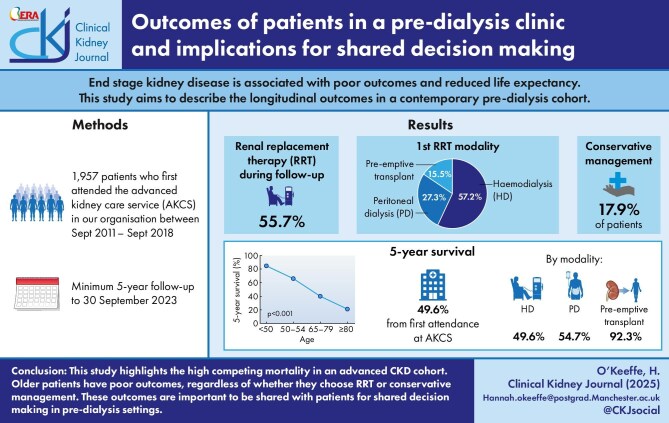

KEY LEARNING POINTS **What was known:**

- End-stage kidney disease (ESKD) is associated with a significantly reduced life expectancy, particularly in older patients.
- Conservative care is an acceptable additional care pathway alongside haemodialysis, peritoneal dialysis and kidney transplantation.
- Patients and caregivers are keen to receive high quality information and participate in shared decision making.

**This study adds:**

- This study describes outcomes in a large contemporary cohort of patients (1957 patients with a median follow-up of 4.9 years) with advanced chronic kidney disease (CKD) in the UK.
- The findings highlight the high competing mortality risk for patients with advanced CKD, and thus the importance of risk factor management and advanced shared decision making.
- The study provides outcomes by modality and age, in particular demonstrating the significantly improved outcomes for those receiving a transplant compared with dialysis, and the poor survival of older patients regardless of modality chosen.

**Potential impact:**

- The results can help to improve healthcare providers understanding of outcomes and improve information shared with people approaching ESKD and their caregivers to facilitate shared decision making conversations.
- The results provide a basis for future work, including the need for improved risk stratification of people with advanced CKD and the ability to predict who will actually reach ESKD, accounting for the competing risk of mortality.

## INTRODUCTION

Patients with advanced chronic kidney disease (CKD) require intensive and specialized management. This is often provided in advanced kidney care or low clearance clinics. The aim of care in these settings includes symptom management, shared decision making regarding renal replacement therapy (RRT) or comprehensive conservative care, preparation for dialysis access and evaluation of suitability for transplantation. Such advanced kidney care clinics have been recognized internationally as providing improved outcomes, and a model of team-based multidisciplinary care is advocated for in the most recent iteration of the Kidney Disease: Improving Global Outcomes (KDIGO) CKD guidance [1–5].

End-stage kidney disease (ESKD) is well recognized as conferring a poor prognosis, with dialysis treatment often associated with a shorter life expectancy than many malignancies [6, 7]. Indeed, 5 years after commencing haemodialysis (HD), just 42% of patients in the USA and 50% of patients in Australia and New Zealand are alive [8, 9]. In the UK, the 5-year survival of patients who commenced RRT (any modality) between 2012 and 2015 was 71%–73% for those under 65 years, but just 30%–35% for those older than 65 years [10]. Non-dialysis dependent CKD is also associated with an increased risk of mortality, with this risk increasing with lower levels of renal function [11, 12]. Due to the high cardiovascular risk profile of those with CKD and the competing risk of mortality, many patients with advanced kidney disease do not survive to the commencement of dialysis even after choosing this trajectory of care.

In particular, elderly patients have been reported as having approximately double the mortality of younger dialysis patients. Wachterman *et al*. reported 22.5% of Medicare beneficiaries over 65 years of age initiating dialysis died within 30 days, 44.2% within 6 months and 54.5% within 1 year [13]. In elderly and multimorbid patients, comprehensive conservative care or best medical management may be the preferred option if RRT is unlikely to improve their symptom burden or quality of life, or confer a survival advantage [14–18]. Some studies have shown that while dialysis may increase survival time, conservative care can offer increased hospital-free time and improved quality of life [18–20].

When counselling patients, an ability to prognosticate helps to frame the options for patients and caregivers and aid with decision making, with evidence supporting that patients wish to receive this information [14, 21–23]. Despite this, the literature suggests that good quality shared decision making remains poorly implemented in clinical practice [14, 24].

To better understand the clinical trajectory of patients with advanced CKD and how this might impact shared decision making, this study aims to describe the long-term outcomes of a pre-dialysis patient cohort, specifically evaluating mortality outcomes across age groups, different dialysis modalities and conservative care.

## MATERIALS AND METHODS

### Study design and setting

We present longitudinal follow-up of a patient cohort from our multidisciplinary Advanced Kidney Care Service (AKCS) clinic based in Salford Royal Hospital, a large tertiary renal centre in the Northern Care Alliance NHS Foundation Trust, UK, with a catchment population of 1.55 million. Patients aged ≥18 years are routinely referred to this clinic once their outpatient estimated glomerular filtration rate (eGFR) falls to <20 mL/min/1.73 m^2^. The AKCS clinic provides comprehensive care, delivered by a specialist multidisciplinary team (including doctors, specialist nurses and dietitians), who provide education and support for shared decision making around modality choice and dialysis access planning. Specialist CKD nurse home visits are routinely offered to educate on lifestyle changes and RRT options, and encourage home therapies for patients. Potential treatment modalities are typically discussed by the specialist nurses at these visits as well as at the patients’ AKCS appointments. The aim is to make a shared decision on the best modality for the individual including consideration of patient factors, comorbidities, frailty, patient and carer preferences, lifestyle and beliefs. These discussions begin once the patient enters the AKCS clinics. These decisions are dynamic and may change over time. Early transplant evaluation is conducted in suitable individuals with the aim of achieving pre-emptive transplantation. Patients are referred to the renal psychology team for support when needed. The decision to commence RRT is based on discussion with patients and takes into account symptoms, blood results and fluid balance, rather than at a set eGFR threshold. Although there is not a fixed eGFR threshold, patients typically commence RRT when their eGFR is between 6 and 10 mL/min/1.73 m^2^, depending on those other factors.

### Data collection

**Figure 1:**
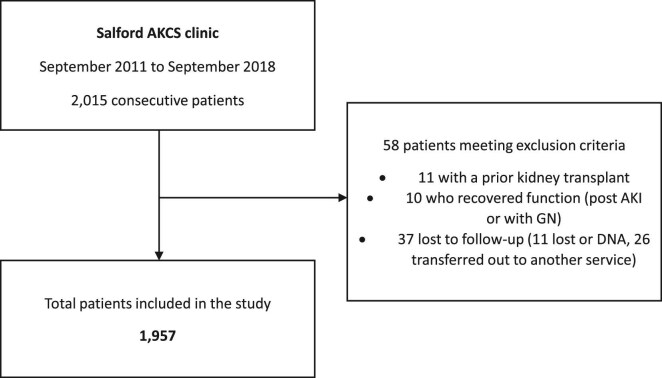
Study consort diagram. AKI, acute kidney injury; GN, glomerulonephritis; DNA, did not attend.

Data was collected using the organization's electronic patient record. All consecutive patients who first attended the AKCS clinic from September 2011 through to September 2018 were reviewed, with a minimum of 5 years of follow-up through to 30 September 2023. Data collected included patient demographics, cause of CKD, comorbidities including a history of diabetes mellitus, hypertension and cardiovascular disease (including coronary artery disease and heart failure), date of first attendance in AKCS, eGFR and urine albumin:creatinine ratio (uACR) at first attendance, date of first RRT, modality of RRT, decisions regarding active versus conservative care and death. Socioeconomic status was determined using the online Ministries of Housing, Communities and Local Government tool (available at https://imd-by-postcode.opendatacommunities.org/imd/2019) which enables uploading of postcodes to get the corresponding 2019 indices of deprivation [25]. Patients were grouped into quintiles based on relative deprivation, with quintile 1 being the most deprived and quintile 5 the least. Where urine protein:creatinine ratio only was available, uACR was calculated from this [26]. A total of 2015 patients initiated care in AKCS during these 8 years (Fig. 1). Patients with a prior kidney transplant, those who recovered renal function and were discharged from the AKCS clinic, and those who were lost to follow-up were excluded, leaving 1957 patients for inclusion in this study. Patients who were documented in AKCS clinic letters as choosing conservative care were coded as being on the conservative pathway, with the others as being on an RRT or active pathway.

### Statistical analysis

Analyses were performed using IBM SPSS version 29 software registered with the University of Manchester. Continuous variables were expressed as median and interquartile range (IQR) after checking for normality of the distribution. Categorical variables were expressed as number and percentage. Kaplan–Meier curves were used to demonstrate the survival difference between patient groups. Cox regression was used to calculate the hazard ratio (HR) for mortality between groups, including for age groups and comorbidities. A *P*-value of <.05 was taken as being statistically significant throughout the analyses. The baseline demographics of the cohort are described, as well as the number progressing to RRT, and the initial RRT modalities in the cohort. Overall mortality is described, as well as mortality stratified by age group and according to RRT modality or conservative care. For patients who opted for conservative care, a timepoint of when they reached an eGFR of <10 mL/min/1.73 m^2^ on two consecutive tests was used as a comparator to the date of RRT commencement in some of the survival analyses to exclude transient reductions in function.

### Ethical approval

This study was prospectively registered with the Northern Care Alliance's Research and Innovation Committee (Reference 24HIP14). Full ethics review was not deemed to be required as the study utilized retrospective anonymized data collected as part of routine patient care, and the need for individual patient consent was waived by the committee.

## RESULTS

The baseline characteristics of the overall cohort are presented in Table 1. The median follow-up was 4.9 years (IQR 2.1–7.7). The median age was 69 years, and 42% of the cohort were female. At first clinic visit, the median eGFR was 16 mL/min/1.73 m^2^, and uACR was 31.7 mg/mmol. The cohort was predominantly of White ethnicity (82%) with Asian ethnicities accounting for 13%. All patients were documented as having hypertension (100%), a significant proportion had diabetes (44.9%) and almost a quarter had cardiovascular disease (22.7%). Diabetes was the most common cause of CKD in 31.9% of the cohort, followed by hypertension or renovascular disease at 14.7%, glomerulonephritis in 7.5%, autosomal dominant polycystic kidney disease in 6.1%, obstructive or urological causes in 6.1%, and other causes or an unspecified aetiology accounting for the remaining 33.6%. There were more patients with lower Index of Multiple Deprivation (IMD) status in the cohort: 42.9% (*n* = 839) in IMD quintile 1, 19% (*n* = 372) in 2, 12.3% (*n* = 240) in 3, 14.2% (*n* = 277) in 4 and 9.4% (*n* = 183) in 5. IMD data were missing for the remaining 2.4%% (*n* = 46).

**Table 1:** Baseline demographics of the study cohort, those who opted for RRT and those who opted for conservative management.

Variable	Overall cohort, *n* = 1957	Opted for RRT, *n* = 1607 (82.1%)	Opted for conservative, *n* = 350 (17.9%)	*P*-value
Age, years	69 (56.9–78.1)	66.1 (54.1–74.6)	81.8 (76.8–85.7)	**<.001**
Female, % (*n*)	42 (821)	40.1 (645)	50.3 (176)	**<.001**
Baseline eGFR, mL/min/1.73 m^2^	16 (12–18)	15 (12–18)	16 (13–19)	**<.001**
Baseline uACR, mg/mmol	31.7 (5.8–128.0)	36.6 (6.6–144.6)	13.4 (5.1–58.9)	**<.001**
BMI, kg/m^2^	28.2 (24.2–32.8)	28.5 (24.4–33.3)	27.2 (22.9–31.0)	**.011**
Smoking, % (*n*)				**<.001**
Never	32.7 (647)	36.2 (582)	18.6 (65)	
Current	10.3 (204)	11.6 (187)	4.9 (17)	
Previous	21.8 (430)	22.7 (364)	18.9 (66)	
Unknown	35.2 (695)	29.5 (474)	57.7 (202)	
Ethnicity, % (*n*)				.071
White	82.3 (1610)	81.4 (1308)	86.3 (302)	
Asian	13.4 (263)	14.3 (230)	9.4 (33)	
Black	1.6 (31)	1.7 (28)	0.9 (3)	
Mixed or other	2.7 (53)	2.6 (41)	3.5 (12)	
IMD Decile, % (*n*)				.492
1	42.9 (839)	43.1 (693)	41.7 (146)	
2	19 (372)	18.5 (298)	21.7 (76)	
3	12.3 (240)	12.7 (204)	10 (35)	
4	14.2 (277)	14.2 (228)	14 (49)	
5	9.4 (183)	9.3 (150)	9.7 (34)	
Missing	2.4 (46)	2.1 (34)	2.9 (10)	
Comorbidities, % (*n*)				
Diabetes	44.9 (879)	44.7 (719)	45.7 (160)	.748
Hypertension	100 (1957)	100 (1607)	100 (350)	n/a
Cardiovascular disease	22.7 (444)	17.8 (286)	30.0 (105)	**.024**
Cause of CKD, % (*n*)				**<.001**
Diabetes	31.9 (625)	33.4 (536)	25.4 (89)	
Hypertension/renovascular	14.7 (288)	12.9 (208)	22.9 (80)	
Glomerulonephritis	7.4 (146)	8.5 (136)	2.9 (10)	
Polycystic kidney disease	6.1 (120)	7.3 (118)	0.6 (2)	
Obstructive/urological	6.1 (120)	5.4 (86)	9.7 (34)	
Other causes/not specified	33.6 (658)	32.5 (523)	38.6 (135)	
Medications, % (*n*)				
ACEi or ARB	46.2 (905)	47.5 (763)	40.6 (142)	**.019**
Statin	60.1 (1177)	59.5 (956)	63.1 (221)	.210
Antiplatelet	38.5 (754)	36.8 (592)	46.1 (162)	**.001**
Baseline blood results				
Haemoglobin, g/L	112 (102–121)	111 (102–121)	112 (103–120)	.905
Calcium, mmol/L	2.2 (2.1–2.3)	2.2 (2.1–2.3)	2.2 (2.1–2.3)	**.018**
Phosphate, mmol/L	1.3 (1.1–1.5)	1.3 (1.1–1.6)	1.2 (1.1–1.4)	**<.001**
Bicarbonate, mmol/L	21.4 (18.4–24.6)	21.6 (18.6–24.7)	20.2 (18.1–23.9)	.174
Albumin, g/L	41 (38–44)	41 (38–44)	41 (39–43)	.781
PTH, ng/L (ref range 15–65)	29.5 (14.7–92.6)	29.3 (14.9–96.2)	30.9 (13.7–85.6)	.375

Continuous values are presented as median with IQR and *P*-value by Mann–Whitney U test, and categorical variables as percentage and number, and *P*-value by Chi-Square test.

Significant *P*-values are indicated in bold.

BMI, body mass index; ACEi, angiotensin converting enzyme inhibitor; ARB, angiotensin receptor blocker; PTH, parathyroid hormone.

### Renal replacement therapy modalities

**Figure 2:**
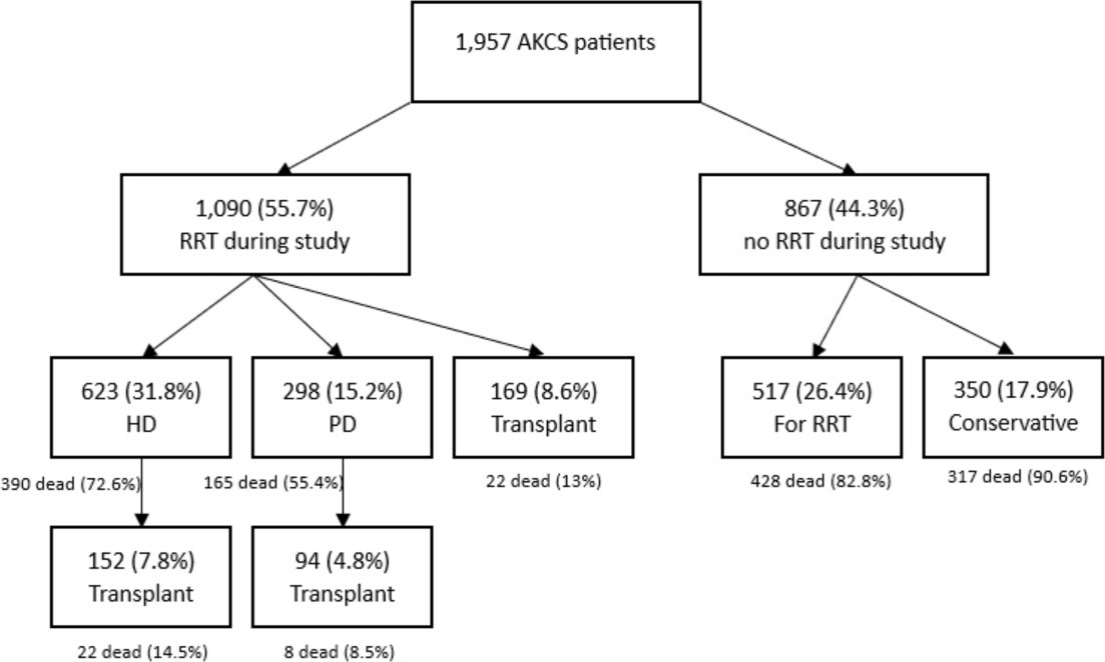
Patient progression to RRT or pathway of care chosen, and patient survival at the end of follow-up in each of these cohorts.

Just over half the cohort (*n* = 1090, 55.7%) initiated RRT during the follow-up period. The median time from first visit to RRT was 13.8 months (IQR 6.0–29.0 months). Of those who progressed to RRT, the first modality was HD in 57.2% (*n* = 623), followed by peritoneal dialysis (PD) in 27.3% (*n* = 298) and a pre-emptive transplant in 15.5% (*n* = 169). A total of 867 patients (44.3% of the overall cohort) did not initiate RRT during the study period, with 350 (17.9% of the overall cohort) having opted for conservative management (Fig. 2).

A total of 415 patients (21.3%) received a transplant at any time during follow-up. Of these, 169 were pre-emptive (first modality in 15.5% of patients commencing RRT) and 246 were following a period of either HD or PD, with 24.4% of HD patients and 31.5% of PD patients receiving a kidney transplant. A total of 126 (30.4%) of the transplants were from live donors and 289 (69.6%) from deceased donors (of which 9 were kidney and pancreas transplants). In those who received a pre-emptive transplant, a higher proportion were from living donors at 42.6% (72/169).

Median patient ages differed in the different modality groups (*P *< .001 by Kruskal–Wallis), with a median age of 63.5 (IQR 53.1–71.9) years in the HD group, 60.8 (46.5–72.3) years in the PD group, 54.7 (43.0–55.1) years in the pre-emptive transplant group, 73.1 (65.2–79.5) years in the active group who did not start RRT and 81.8 (76.8–85.7) years in the conservative care group.

Late presentations, defined as first renal review <90 days prior to RRT start, accounted for 6.5% of this cohort. This will not represent the total late presenters to the department during this period as a large proportion of late presenters will never come to AKCS and will start RRT as an inpatient. This compares to UK renal registry data nationally from the same period of 2011 to 2018, with late presentations varying between 15.6% and 19.6% per annum [27–34].

### Overall mortality

Two thirds of patients (*n* = 1321, 67.5%) died by the end of follow-up. In those that had opted for RRT 122 patients (6.2%) remained alive and had not started RRT during the follow-up period. The overall median survival of the cohort was 5.0 years [95% confidence interval (CI) 4.7–5.3]. The overall 5-year survival was 49.6%. Of the patients who were on the active pathway (*n* = 519) but had not initiated RRT during the study period, 428 (82.5%) died prior to RRT. Patients with diabetes had a HR for death of 1.9 compared with those without (*P *< .001), and those with cardiovascular disease or coronary heart disease had a HR of 2.0 compared with those without (*P *< .001). There was no difference in overall mortality when analysed by IMD quintile (*P *= .079).

**Figure 3:**
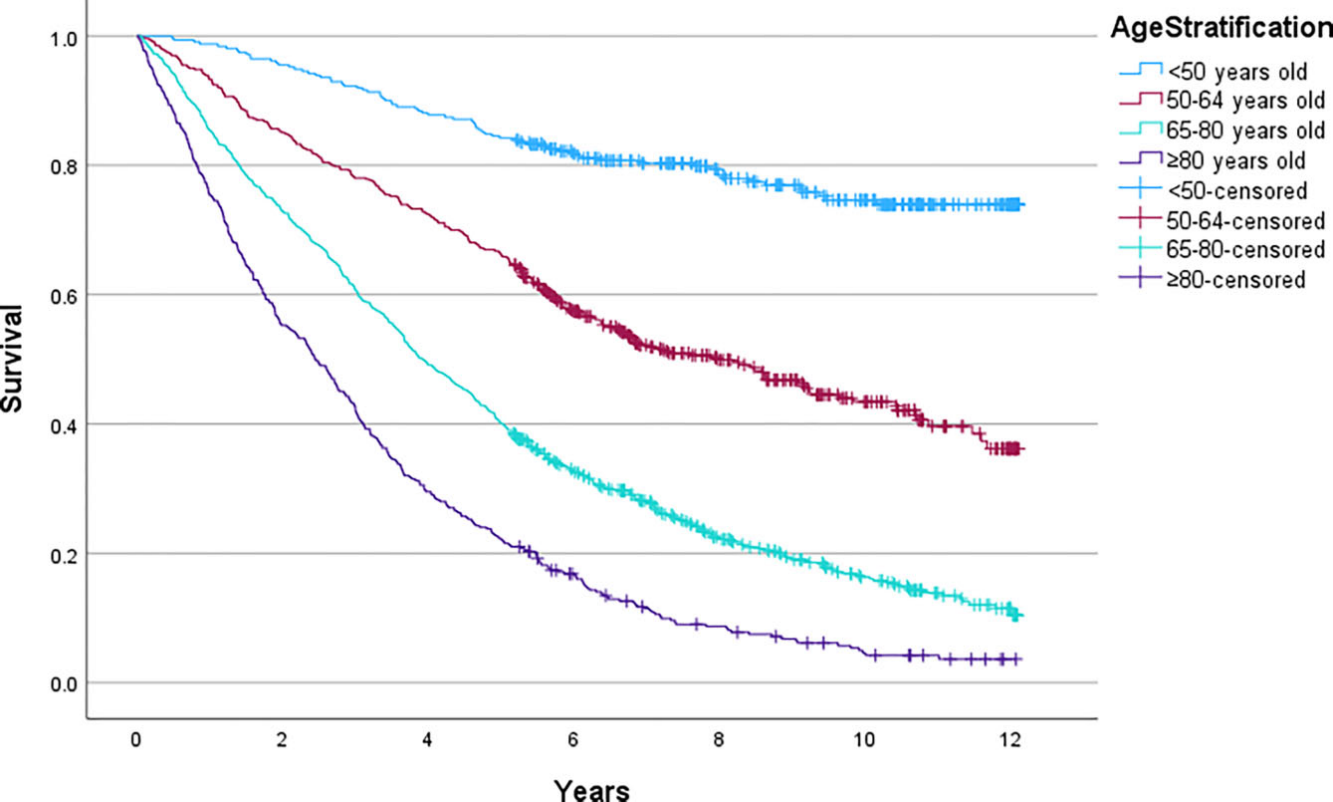
Kaplan–Meier curves showing survival stratified by age <50, 50–64, 65–80 and ≥80 years old.

A significant difference (log-rank *P*-value <.001) in survival was found between younger and older patients with a mean survival of 8.6 years (95% CI 8.3–8.9) for those <65 years old and 5-year survival of 73.4%, compared with a mean survival of 4.4 years (95% CI 4.2–4.6) and 5-year survival of 34.0% for those 65 years or older. When further stratified according to age, the difference in survival remained between younger and older cohorts, as shown in the Kaplan–Meier curve in Fig. 3 (log-rank *P*-value <.001), and with the mean survivals and HRs for mortality shown in Table 2.

**Table 2:** Survival stratified by age.

Age cohort	Mean survival, years (95% CI)	5-year survival (%)	HR for death	*P*-value
<50 years	10.2 (9.8–10.6)	84.2		
50–64 years	7.6 (7.2–8.0)	66.1	3.0	<.001
65–79 years	5.0 (4.7–5.3)	40.1	6.2	<.001
≥80 years	3.3 (3.0–3.6)	22.3	10.5	<.001

### Conservative care outcomes

**Figure 4:**
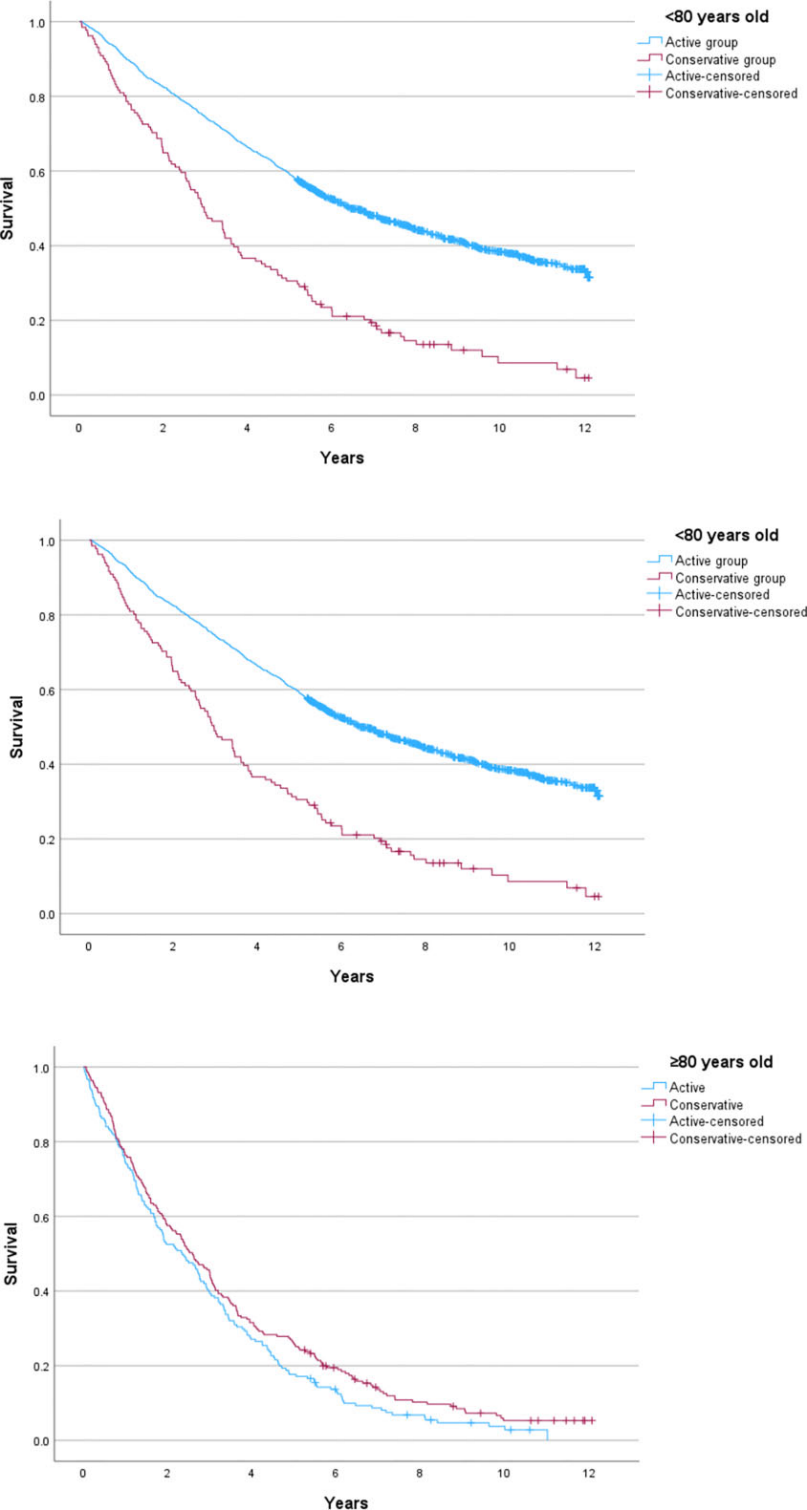
Kaplan–Meier curves showing survival from first ACKS attendance (**A**) by Cox regression, using age as a covariate, (**B**) in the active treatment pathway versus conservative pathway in those <80 years old, and (**C**) in the active treatment pathway versus conservative pathway in those 80 years and older.

Overall, patients who had opted for RRT (including those who received it during the follow-up period and those who were being prepared for but did not initiate it within the follow-up period), had a significantly longer survival than those in the conservative care group (*P *< .001), with median survival of 5.6 years (95% CI 5.2–6.0) and 2.7 years (95% CI 2.4–3.1), respectively, from first AKCS attendance. The conservative care group were older than those who chose to pursue RRT, with a median age of 66.1 years in those opting for RRT (IQR 54.1–74.6) versus 88.1 years in the conservative group (IQR 76.8–85.7). When mortality was adjusted for age, there was no difference between these subgroups, with a HR of 1.1 for the conservative group (95% CI 1.1–1.1) and the relevant Kaplan–Meier curve is shown in Fig. 4A. Figure 4 shows survival from first AKCS attendance for the respective groups. When stratified into age <80 years and age ≥80 years, there was a difference in survival (Fig. 4B and C). For those <80 years, patients in the conservative arm had a median survival of 35.5 months (*n* = 131), compared with 77.0 months (*n* = 1426) in the active group (*P *< .001), with a HR of 2.3 (95% CI 1.9–2.9) for death in the conservative arm. In contrast, for those over 80 years, median survival was 31.1 months (*n* = 219) in the conservative group, versus 28.1 months (*n* = 181) in those opting for the active pathway (*P *= .078). The HR for death in the conservative group was 0.8 (95% CI 0.7–1.0).

**Figure 5:**
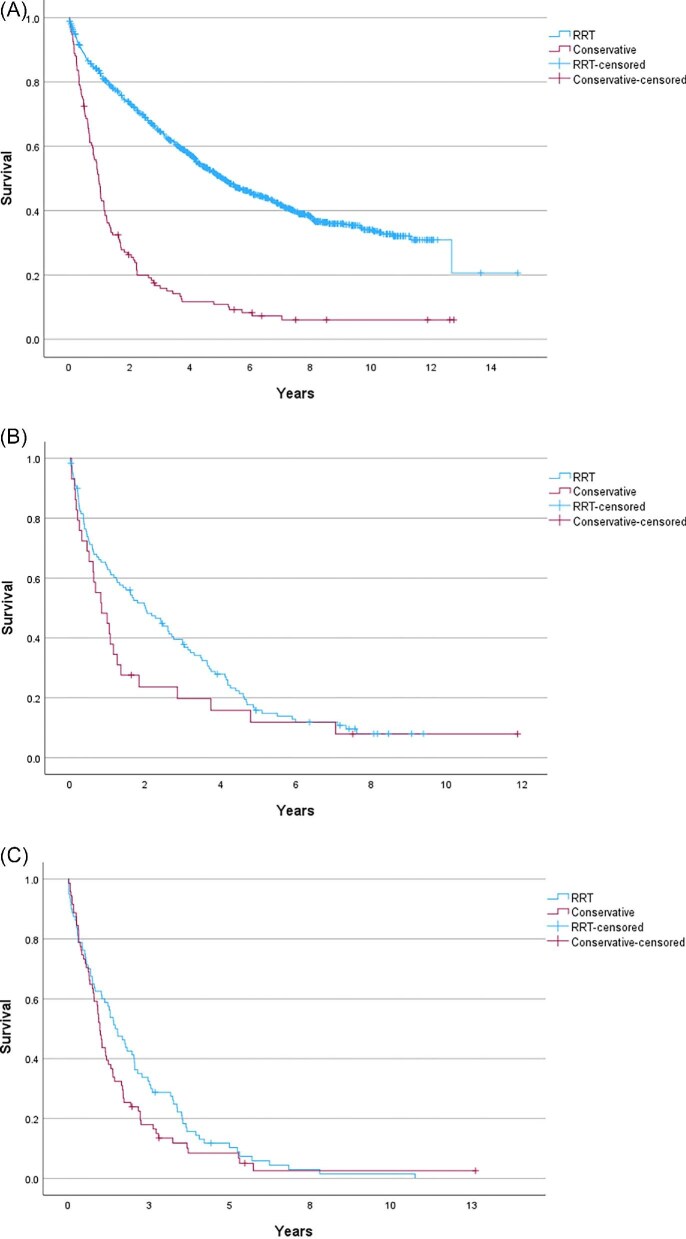
Kaplan–Meier curves showing survival from date of RRT start, compared with the conservative group when eGFR declined to below 10 mL/min/1.73 m^2^ in (**A**) the overall cohort, and (**B**) those aged between 75 and 80 years old, and (C) those over 80.

We compared the survival from date of RRT commencement in those who started RRT, with survival in patients receiving conservative care at the timepoint when their eGFR was <10 mL/min/1.73 m^2^. The results for the overall cohort, and stratified by age are shown in Table 3 and Fig. 5. Figure 5 compares this survival from date of first RRT in the RRT group, versus survival once the eGFR declines below 10 mL/min/1.73 m^2^ in the conservative group.

**Table 3:** Median survival from commencement of RRT.

Age group	Survival from date of RRT (*n* = 1090), months	Survival from eGFR <10 mL/min in those on conservative care (*n* = 139), months	*P*-value
Overall cohort	60.8 (54.2–67.4)	11.9 (10.4–12.1)	**<.001**
70–74 years	32.9 (25.2–40.6)	10.9 (0.0–27.7)	**<.001**
75–79 years	23.9 (15.5–32.4)	10.1 (3.5–16.6)	.055
≥80 years	17.4 (11.8–23.0)	11.8 (10.3–13.2)	**.012**

Age <70 years old not considered due to minimum numbers opting for conservative care.

Significant *P*-values are indicated in bold.

*P*-values by Log Rank.

### Renal replacement therapy outcomes

Those who received a kidney transplant had the best survival (mean of 11.2 years, 95% CI 10.9–11.6; 5-year survival 92.3%), followed by those who had PD (mean 7.5 years, 95% CI 7.0–8.0; median 7.3 years, 95% CI 5.9–8.7; 5-year survival 54.7%), then HD (mean 7.0 years, 95% CI 6.7–7.3; median 6.3 years, 95% CI 5.6–7.0; 5-year survival 49.6%), with a log-rank *P*-value <.001. As <50% of those in the transplant group had died by the end of follow-up it was not possible to calculate their median survival and hence mean survivals are reported for this group and also for the other cohorts to enable comparison.

**Figure 6:**
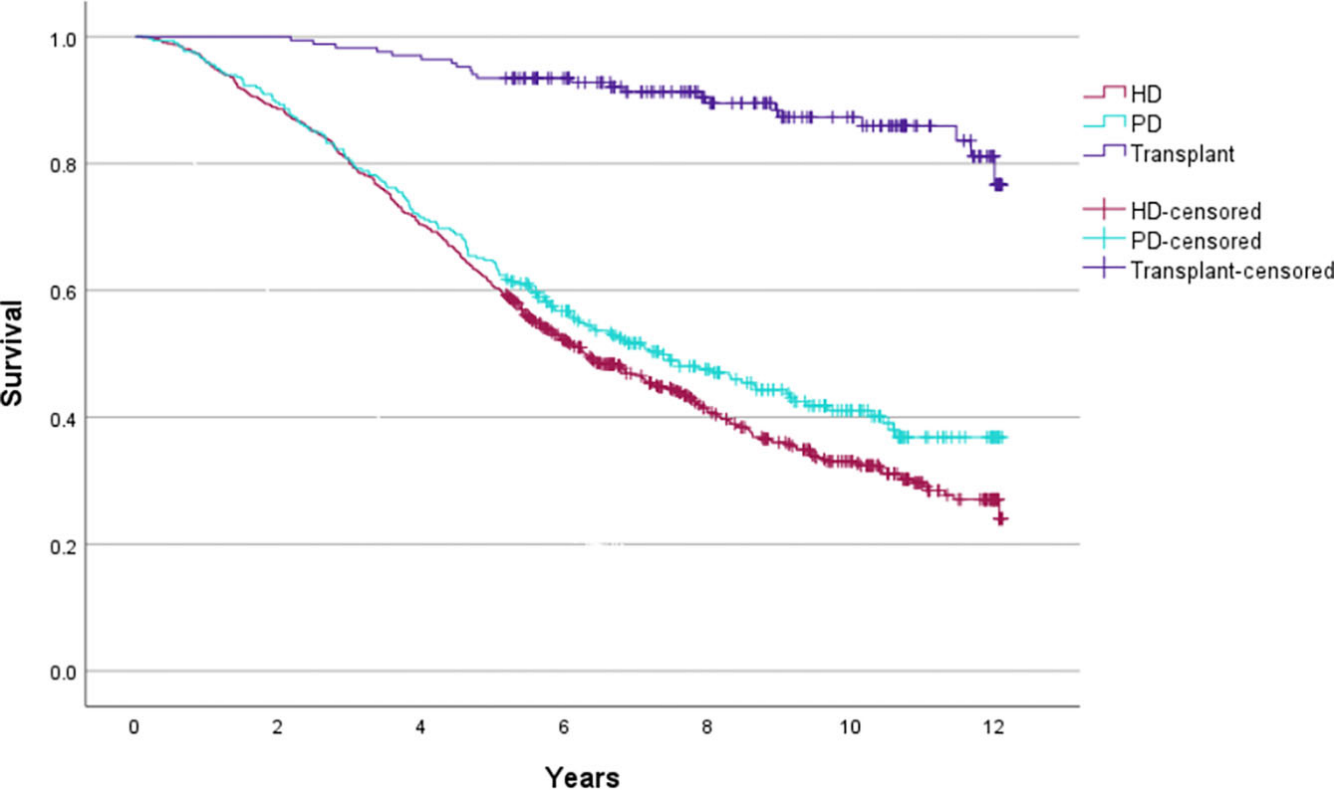
Kaplan–Meier curve showing mortality by modality.

The cumulative mortality for each RRT modality at 30, 90, 180 and 365 days post starting therapy is shown in Table 4 and the Kaplan–Meier curve showing mortality by RRT modality is shown in Fig. 6 (log rank *P*-value <.001). 13.3% of the HD cohort were dead at 1 year, compared with 11.7% of the PD group and 1.2% of the cohort who received a pre-emptive transplant. Cumulative mortality in the conservative group is shown from when their eGFR dropped below 10 mL/min/1.73 m^2^. A total of 39.7% (139/350) of patients in the conservative group reached this eGFR threshold during the follow-up period, and 51.1% (179/350) of patients in the conservative group had died prior to their eGFR declining below 10 mL/min/1.73 m^2^. Once the eGFR was <10 mL/min/1.73 m^2^, 5.8% (8/139) were dead at 30 days, 64% (89/139) at 90 days and 90.6% (126/139) at 180 days.

**Table 4:** Cumulative mortality following the start of each modality.

Number of deaths	HD (*n* = 623)	PD (*n* = 298)	Pre-emptive transplant (*n* = 169)	Conservative care with eGFR <10 (*n* = 139)
At 30 days	9 (1.4)	2 (0.7)	1 (0.6)	8 (5.8)
At 90 days	20 (3.2)	8 (2.7)	1 (0.6)	89 (64.0)
At 180 days	50 (8.1)	22 (7.4)	2 (1.2)	126 (90.6)
At 365 days	83 (13.3)	35 (11.7)	2 (1.2)	126 (90.6)
At 5 years	314 (50.4)	135 (45.3)	13 (7.7)	126 (90.6)

Data are presented as *n* (%).

Log rank *P*-value <.001.

Conservative mortality is from the date when two consecutive eGFRs were <10 mL/min/1.73 m^2^.

Multivariable analysis by Cox regression was performed to assess the outcome by modality adjusting for confounders of age, cardiovascular disease and diabetes. Frailty scores or comorbidity indices, although relevant, were not included as the required data were not available. Consistent with the observed survival in the overall cohort, compared with those who did not receive RRT (conservative care or died before reaching RRT), the HR for death was 0.60 in the HD group (*P *< .001), 0.59 in the PD group (*P *< .001) and 0.13 in the transplant group (*P *< .001).

## DISCUSSION

This study highlights the high mortality faced by patients with advanced CKD and the importance of age as a key determinant of survival. By the end of the follow-up, over two-thirds of patients had died, with the median survival from the first attendance at AKCS clinic being <5 years.

In our cohort, just 55.7% of patients received RRT. This is due to a high proportion of patients opting for conservative medical management (17.9%), in addition to the significant finding that 26.7% of those who had opted for RRT died before reaching the need for RRT. This highlights the extremely high competing mortality in patients with advanced CKD and why these individuals will require personalized holistic management including a focus on addressing cardiovascular risk.

Age is an important factor in survival regardless of the pathway or RRT modality chosen, and this will have implications for clinical decision making. Patients who received RRT had longer survival than those who opted for conservative care, but this survival benefit was reduced for older individuals. Addressing lead-time bias and comparing survival for patients on conservative care is difficult [15]. In an effort to overcome this bias, in addition to assessing survival in both cohorts from first attendance at AKCS, we compared survival from date of first RRT in the actively treated group to the date when two consecutive eGFR readings were <10 mL/min/1.73 m^2^ in the conservatively managed patients. In this analysis, patients aged between 75 and 80 years had a median survival 13.8 months longer from date of RRT commencement compared with those on the conservative pathway whose eGFR had declined <10 mL/min/1.73 m^2^. Comparing the same groups, those older than 80 years who commenced RRT had a median survival just 5.6 months longer than those who opted for conservative care. This does not account for selection bias, with those who actually received RRT likely to have had better functional status and reduced comorbidity burden compared with those on the conservative pathway, which may partially account for the improved survival. Importantly, the observed survival benefit needs to be considered in the context of the burden of RRT, the increased risk of hospitalization, and the impact upon quality of life for these elderly patients [20]. The ‘Prepare for Kidney Care’ prospective randomized controlled trial comparing conservative management to dialysis care is due to report in 2026 and will hopefully serve to address some of the outstanding questions regarding these treatment options in older adults [35].

Reasonable proportions of patients had peritoneal dialysis (27.3%), and a pre-emptive transplant (15.5%) as their first RRT modality. These compare with the national proportions of 18.8% starting RRT with PD, and 6.6% starting with a transplant in the latest UK renal registry report, and highlight the potential benefits of an AKCS strategy in achieving a larger proportion of pre-emptive transplantation or dialysis treatment with PD first [10]. Consistent with previous findings, at 5 years just 50% of those who received HD were still alive and this was 55% for those receiving PD, whilst those receiving a pre-emptive transplant did well, with 92% alive at 5 years [8–10].

Strengths of this study include that it is a descriptive analysis of a large cohort with clearly defined outcomes and followed over a long period of time. Limitations include those of a single-centre study, as well as of a retrospective observational study. The study considers outcomes grouping patients by treatment pathway. Unfortunately, no data on patient's frailty or functional status, and only limited comorbidity data were available for analysis. These factors may contribute to the patient's pathway selection and hence the increased mortality observed in the conservative subgroup. Frailty and comorbidity burden are clinically important factors which may impact treatment pathway choice, modality choice and mortality [19, 36–38]. Frailty is now being captured prospectively in our AKCS from first attendance. The high mortality in the conservative group likely reflects a degree of selection bias, with patients opting for conservative care in practice potentially more likely to have increased frailty, comorbidities or a reduced life expectancy [39].

In conclusion, a significant competing risk of mortality exists in patients with advanced CKD, and this increases progressively with increasing age. It is important to educate and inform patients, carers and healthcare workers, about expected outcomes with different options including RRT modalities and conservative care. Improved personalized estimates of risk of progression to ESKD and of the competing risk of mortality are required to help inform individualized patient-centred, shared decision making, especially in highly comorbid elderly patients. Further research in this important area in nephrology is required. There is no doubt that many elderly patients with frailty and significant comorbidities commence RRT but do not benefit symptomatically or survive longer, and suffer a reduced quality of life related to the burden and complications of dialysis treatment. An improved understanding of the factors contributing to the risk of mortality in patients with advanced kidney disease, combined with the corresponding development and validation of risk-stratification tools in this vulnerable group of patients could facilitate more meaningful decision making for future care.

## Data Availability

The data underlying this article will be shared on reasonable request to the corresponding author.
